# Comparison of various steady state surrogate insulin resistance indices in diagnosing metabolic syndrome

**DOI:** 10.1186/s13098-019-0439-5

**Published:** 2019-06-14

**Authors:** Sikandar Hayat Khan, Ali Nawaz Khan, Nayyer Chaudhry, Roomana Anwar, Nadeem Fazal, Muhammad Tariq

**Affiliations:** 1 Department of Pathology PNS HAFEEZ, Islamabad, Pakistan; 2 Armed Forces Institute of Cardiology, Rawalpindi, Pakistan; 3 Department of Chemical Pathology (AFIP), Rawalpindi, Pakistan; 4 Department of Biochemistry, Baqai Medical University, Rawalpindi, Pakistan; 5 Department of Medicine PNS HAFEEZ, Islamabad, Pakistan; 6 Healthcare Administration PNS HAFEEZ, Islamabad, Pakistan

**Keywords:** Insulin resistance, HOMAIR, HOMA2, QUICKI, G/I ratio, Fasting Insulin Resistance Index (FIRI), Metabolic syndrome

## Abstract

**Background:**

Insulin resistance is core cause of metabolic syndrome. Determining insulin resistance is one of the foremost requirements imperative to understanding the pathophysiology of disease. The gold standard “Euglycaemic clamp test” is cumbersome, long and non-feasible in routine clinical setups to diagnose metabolic syndrome. Various continuous and steady state insulin resistance indices are now available in literature. We plan to evaluate commonly utilized steady state insulin resistance indices directly and Homeostasis Model Assessment for Insulin Resistance (HOMAIR) with added triglyceride (HOMA-TG index).

**Methods:**

The cross-sectional study was carried from Jan-2016 to Dec-2018 at PNS HAFEEZ and department of chemical pathology, AFIP with following objectives: (1) To evaluate steady state insulin resistance markers for diagnosing metabolic syndrome as per IDF defined criteria by ROC curve analysis, (2) to measure Kendal Concordance between various insulin resistance indices and (3) to correlate steady state insulin resistance markers with anthropometric and lipid indices. After several exclusions we selected 224 subjects based upon “non-probability convenience sampling” for inclusion in study. Clinical history, anthropometric measures were calculated and sampling was done for insulin, glucose and other biochemical parameters. Metabolic syndrome was diagnosed as per IDF criteria, while HbA1c was utilized to diagnose diabetes mellitus. Pearson correlation was used to correlate various steady state insulin resistance indices including HOMAIR, HOMA2 index, QUICKI, G/I ratio, HOMA-TG index and serum insulin. AUC was calculated by ROC analysis for all surrogate insulin measures in diagnosis of metabolic syndrome.

**Results:**

“HOMA-TG index” has shown the highest AUC for diagnosing metabolic syndrome along with higher correlation with lipid markers and anthropometric indices in comparison to other steady-state insulin resistance markers. Furthermore, QUICKI and G/I ratio showed the lowest AUC for detection of metabolic syndrome.

**Conclusion:**

“HOMA-TG index” has shown highest AUC for metabolic syndrome diagnosis. However, QUICKI and G/I ration showed the lowest AUC for detection of metabolic syndrome. It is hoped that the potential “HOMA-TG index” may provide better diagnostic efficiency for diagnosing metabolic syndrome.

## Background

Insulin resistance has been considered as a major determinant of various metabolic clusters, which overtime have been given various names like “Syndrome X”, “Reaven syndrome” and recently as “Metabolic syndrome” [1]. While the entity seems to omnipresent in every society around the world, still there are no universal criteria defining it [2–4]. This syndrome definition in general includes certain anthropometric measures and biochemical parameters with a scoring system to label someone as having the disease or otherwise. The disordered metabolic pathways underlying the overall entity are lined with massive morbidity and mortality due to atherosclerotic cardiovascular disease (ASCVD) and other metabolic ailments like polycystic ovarian syndrome (PCOS) and non-alcoholic stetohepatitis (NASH) [5]. While there are variable definitions of metabolic syndrome so compelling need is there to develop a definitive biomarker to conclude its presence. In this regard the gold standard technique “Euglycaemic clamp test” is considered to be very useful but not feasible and difficult to deploy in busy clinics [6]. Therefore, there was a clear need established to develop surrogate insulin resistance marker for routine clinical use to identify a biochemical measure to diagnose and monitor subjects with metabolic syndrome [7].

Currently, three types of surrogate insulin resistance markers are available to diagnose [7, 8]. First type includes investigations which involve glucose loading and the measuring glucose and insulin alongside. The long continuous or regularly sampled continuous insulin infusion techniques include the aforementioned euglycaemic clamp test, Matsuda index where insulin is administered, frequently sampled glucose only methods like oral glucose tolerance test (OGTT), and glucose and insulin combined sampling techniques like “minimally model analysis of frequently sampled intravenous glucose tolerance test (MINMOD), Insulinogenic index (IGI), frequently sampled intravenous glucose tolerance test (FSIVGTT)” [6–8]. The second category consist of markers which addresses insulin resistance in steady-state without any glucose or other intervention. The list her includes Homeostasis Model Assessment for Insulin Resistance (HOMAIR), HOMA2 index, glucose insulin ratio (GI ratio), Quantitative Insulin Sensitivity Check Index (QUICKI), Fasting Insulin Resistance Index (FIRI) [8, 9]. The last category include indirect markers which do not use insulin or glucose but newer markers which the literature search has identified to be associated with insulin resistance like ferritin, insulin growth factor binding protein-1, adiponectin, resistin, and some other chemical compounds [8, 10].

The most determined and least clinically utilized role of lipid metabolism in relationship with insulin resistance needs definitive clinical translation. Nor only that lipids, and in specific triglycerides expands the lipid stores/fat deposition through stimulation of lipoprotein lipase but also causes increase in lipolysis leading to hypertriglyceridemia [11]. The criteria of metabolic syndrome already acknowledges there but most of the reviewed data does not incorporate the effect of lipids in mathematical equations of surrogate insulin resistance biomarkers [3, 4]. Some studies have indirectly included lipids, especially triglycerides in association with other biochemical parameters to generate valuable new mathematical indicators for insulin resistance, albeit with differing results [12–14]. Why not insulin with triglycerides? The authors feel that this aspect has to be considered in relation with in vogue steady state clinical biomarkers in some way.

Considering multiple steady-state insulin resistance biomarkers we decided to compare them through ROC curve analysis. We also decided to study the concordance between these steady state markers. We also planned to correlate these steady-state insulin resistance indices for glycated hemoglobin, anthropometric and lipid indices.

## Subjects and methods

### Study settings and design

The setting of study was mainly pathology and medicine departments at Naval Hospital Islamabad in collaboration with department of chemical pathology (AFIP). The study was comparative cross-sectional and was conducted from Jan-2016 to Dec-2018. The study was started after formal approval of hospital authorities and the received signed ethical review committee approval on completion based upon “non-probability convenience sampling”.

### Study subjects

The adult subjects with age more than 18 years who came to hospital for routine medical check-up at the department of medicine were asked for volunteering as study participants. The major exclusion were individuals who had known diabetes, hypertension, chronic disease, diabetics on treatments, old (age > 70 years) or having any associated acute or chronic medical or surgical disorder were not included in the study.

### Measurements and analysis

The participants were asked to report in “Exact medical fasting status” after explaining to them the fasting requirements to report in pathology department on a given working day. Those participants who visited lab at the given time (08:00 to 09:00) were explained in detail about the research project and sampling needs. Finally volunteering subjects were asked to sign a “written consent form” for inclusion into study. Following that the participants were evaluated through clinical history and medically examined for possibility of any chronic disease signs. The anthropometric was also measured for weight, height, waist and hip circumference [15]. We collected up to 10 ml of blood from all participants (n = 232) for fasting plasma glucose, HbA1c, insulin, lipid parameters. Total cholesterol was measured by CHOD–PAP methodology while triglycerides and glucose analyzed by GPO–PAP and GOD–PAP techniques. Glycated hemoglobin was analyzed by ion exchange resin chromatography technique. High density lipoprotein cholesterol (HDLc) and low density lipoprotein-cholesterol (LDLc) were analyzed using cholesterol esterase method on clinical chemistry system (AVIDA 1800. Insulin was analyzed by chemi-luminescence method on immunoassay analyzer (Immulite^®^ 1000). The details of steady state insulin resistance measures are described in Table 1.

**Table 1:** List of various steady-state insulin resistance indices with formula and references

Steady-state insulin resistance marker	Equation	References
Serum insulin	–	–
Homeostasis Model Assessment for Insulin Resistance (HOMAIR)	HOMAIR = fasting insulin × fasting plasma glucose/22.5	[16]
HOMA2 index	Online HOMA2 calculator	HOMA2 Calculator downloaded from https://www.dtu.ox.ac.uk/homacalculator/download.php. Retrieved on: 24-March-2019
Glucose insulin ratio (GI ratio)	GI ratio = fasting plasma glucose/serum insulin	[17]
Quantitative Insulin Sensitivity Check Index (QUICKI)	QUICKI = 1/[Log (insulin μU/ml) + Log (glucose mg/dl)]	[8]
Fasting Insulin Resistance Index (FIRI)	FIRI = fasting insulin × fasting plasma glucose/25	[8]

IDF criteria was utilized to categorize subjects with metabolic syndrome [18]. Subjects having Hba1c less than 6.5% were cauterized as not having diabetes, while above that were diagnosed to have diabetes. We proposed a combine “HOMA-triglyceride index” (HOMA-TG index) to allow the combined assessment of insulin resistance with triglycerides.

The hospital laboratory is a member of “National External Quality Assurance Program Pakistan (NEQAPP)”. An attempt is always made to conform external and internal QC targets in terms of both precision and accuracy with the help of Westgard’s rule and any errors in this regard are documented and addressed. Current inter-bath coefficient of variability (% CV) for triglyceride is less than 2.5% (1.5–2.5%) and while it is between 4 and 5% in routine. Within batch CV% is < 1.5% for triglycerides and < 3.0% for insulin.

We lost eight samples during analysis due to multiple reasons including hemolysis (n = 3), quantity not sufficient (n = 3) and chylous samples (n = 2). We also lost one sample where the technician failed to perform LDLc and HDLc due to some technical issue. These patients were recalled but unlike other patients they were lost to follow up.

### Data analysis

The data was evaluated through IBM SPSS version 19. The descriptive statistics for various steady-state insulin resistance were calculated through SPSS for mean and standard deviation. Frequency of age and gender was also calculated through SPSS descriptive statistics. We utilized Pearson’s correlation to study various surrogate insulin resistance markers with lipid and anthropometric indices. Non-parametric independent sample-test (Mann–Whitney U test) was utilized to compare the differences of various surrogate insulin resistance indices in subjects with or without diabetes. We also utilized “Related sample Kendall’s Coefficient” to study concordance between various steady state insulin resistance measures in a pair wise manner. Finally area under curve (AUC) was calculated by using receiver operative curve (ROC) analysis for various steady-state surrogate insulin resistance markers in diagnosing metabolic syndrome.

## Results

**Fig. 1:**
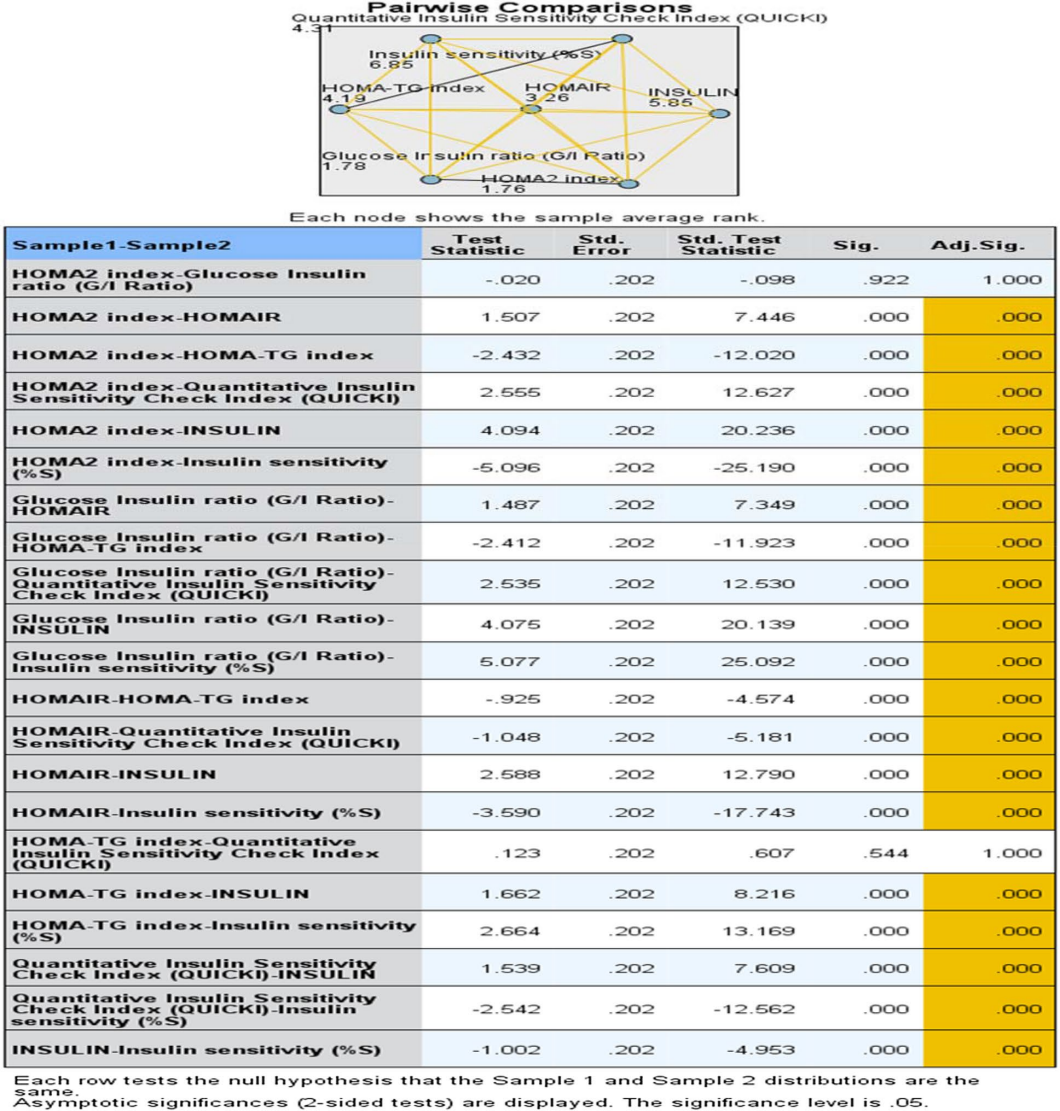
Related sample Kendall’s coefficient of concordance along with pairwise comparison of various steady state insulin resistance measures (p < 0.05)

**Fig. 2:**
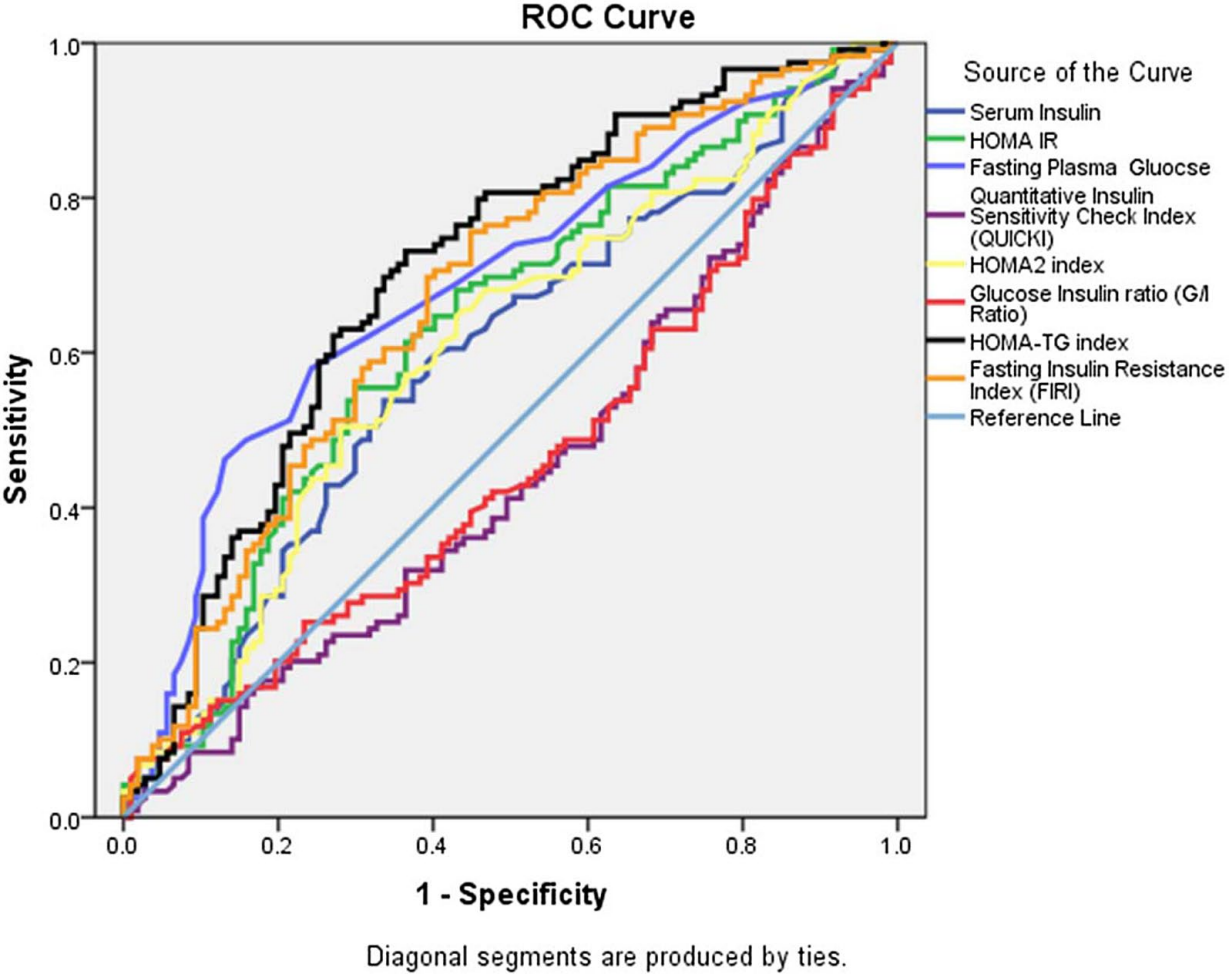
ROC showing area under curve (AUC) for various steady state surrogate markers for IDF-defined metabolic syndrome

We had a total of 224 subjects included in analysis with 52.6% females and 57.7 males. Average age in our data set was 46.56 ± 11.94. Pearson’s correlation between various surrogate steady-state insulin resistance measures and lipid indices are shown in Table 2 with better correlations depicted for HOMA-TG index and FIRI. Table 3 shows correlation between surrogate insulin measures and anthropometric indices where we observed that FITI, HOMA-TG index and both HOMA indices showed higher readings than QUICKI and G/I ratio. Independent sample t-statistics demonstrated only HOMAIR and HOMA-TG index to be statistically different between groups with or without diabetes mellitus (Table 4). Figure 1 shows related sample “Kendall’s Coefficient of Concordance” along with pairwise comparison for various insulin resistance measures (p < 0.05). Area under curve (AUC) for various steady state surrogate markers for insulin resistance in diagnosing metabolic syndrome was highest for HOMA-TG index [0.706 (95% CI 0.638–0.775), p < 0.001)], followed by fasting plasma glucose [0.690 (95% CI 0.621638–0.759), p < 0.001)], FIRI [0.674 (95% CI 0.604–0.745), p < 0.001)], HOMAIR [0.632 (95% CI 0.559–0.705), p = 0.001)], HOMA2 index [0.608 (95% CI 0.535–0.682), p = 0.005)] and serum insulin [0.595 (95% CI 0.521–0.670), p = 0.013)]. QUICKI and G/I ratio demonstrated lowest AUC with former as [0.449 (95% CI 0.374–0.524), p = 0.185)] and later showing [0.462 (95% CI 0.386–0.537), p = 0.332)] (Fig. 2).

**Table 2:** Pearson’s correlation between various surrogate insulin resistance measures and lipid indices

Insulin resistance surrogate markers	Total cholesterol	Fasting triglyceride	HDLc	LDLc	Non-HDLc
Serum insulin					
Pearson correlation	0.091	0.169*	− 0.068	0.001	0.109
Sig. (2-tailed)	0.169	0.010	0.310	0.989	0.102
N	224	224	223	223	223
Homeostasis Model Assessment for Insulin Resistance (HOMAIR)					
Pearson correlation	0.097	0.290**	− 0.085	− 0.035	0.125
Sig. (2-tailed)	0.146	< 0.001	0.199	0.598	0.060
N	224	224	223	223	223
Quantitative Insulin Sensitivity Check Index (QUICKI)					
Pearson correlation	− 0.066	− 0.139*	0.091	0.020	− 0.116
Sig. (2-tailed)	0.316	0.034	0.167	0.763	0.078
N	224	224	223	223	223
Homeostasis Model Assessment 2 index (HOMA2 index)					
Pearson correlation	0.069	0.224**	− 0.085	0.002	0.087
Sig. (2-tailed)	0.292	0.001	0.199	0.982	0.186
N	224	224	223	223	223
Glucose insulin ratio (G/I Ratio)					
Pearson correlation	− 0.084	− 0.098	0.057	− 0.011	− 0.090
Sig. (2-tailed)	0.201	0.136	0.393	0.873	0.172
N	224	224	223	223	223
Homeostasis Model Assessment-Triglyceride index (HOMA-TG index)					
Pearson correlation	0.187**	0.455**	− 0.095	− 0.091	0.208**
Sig. (2-tailed)	0.004	< 0.001	0.152	0.171	< 0.001
N	224	224	223	223	223
Fasting Insulin Resistance Index (FIRI)					
Pearson correlation	0.237**	0.498**	− 0.115	− 0.048	0.256**
Sig. (2-tailed)	< 0.001	< 0.001	0.082	0.468	< 0.001
N	224	224	223	223	223

* < 0.05

** < 0.01

**Table 3:** Pearson’s correlation between anthropometric measures and insulin resistance

Insulin resistance surrogate markers	BMI	WHpR	WHtR
Serum insulin			
Pearson correlation	0.128	0.154*	0.234**
Sig. (2-tailed)	0.054	0.020	< 0.001
N	224	224	224
Homeostasis Model Assessment for Insulin Resistance (HOMAIR)			
Pearson correlation	0.126	0.155*	0.260**
Sig. (2-tailed)	0.058	0.019	< 0.001
N	224	224	224
Quantitative Insulin Sensitivity Check Index (QUICKI)			
Pearson correlation	− 0.104	− 0.050	− 0.082
Sig. (2-tailed)	0.113	0.450	0.213
N	224	224	224
Homeostasis Model Assessment 2 index (HOMA2 index)			
Pearson correlation	0.145*	0.140*	0.239**
Sig. (2-tailed)	0.027	0.034	< 0.001
N	224	224	224
Homeostasis Model Assessment-Triglyceride index (HOMA-TG index)			
Pearson correlation	0.107	0.135*	0.233**
Sig. (2-tailed)	0.104	0.041	< 0.001
N	224	224	224
Fasting Insulin Resistance Index (FIRI)			
Pearson correlation	0.142*	0.188**	0.281**
Sig. (2-tailed)	0.031	0.004	< 0.001
N	224	224	224
Glucose insulin ratio (G/I ratio)			
Pearson correlation	− 0.191**	− 0.078	− 0.163*
Sig. (2-tailed)	0.003	0.236	0.013
N	224	224	224

*BMI* body mass index, *WHpR* waist to hip ratio, *WHtR* waist to height ratio

* < 0.05

** < 0.01

**Table 4:** Differences of various steady state surrogate insulin resistance markers in subjects with or without diabetes mellitus by using non-parametric assumptions (Mann–Whitney U test)

Insulin resistance surrogate markers	Diabetes diagnosis based upon HbA1c	N	Mean rank	Sum of ranks	Sig. (2-tailed)
Serum insulin	HbA1c < 6.50%	178	110.40	19,650.50	0.339
	HbA1c > 6.50%	46	120.64	5549.50	
	Total	224			
Homeostasis Model Assessment for Insulin Resistance (HOMAIR)	HbA1c < 6.50%	178	106.31	18,924.00	*0.005*
	HbA1c > 6.50%	46	136.43	6276.00	
	Total	224			
Quantitative Insulin Sensitivity Check Index (QUICKI)	HbA1c < 6.50%	178	109.95	19,571.00	0.247
	HbA1c > 6.50%	46	122.37	5629.00	
	Total	224			
Homeostasis Model Assessment 2 index (HOMA2 index)	HbA1c < 6.50%	178	108.61	19,333.00	0.077
	HbA1c > 6.50%	46	127.54	5867.00	
	Total	224			
Fasting Insulin Resistance Index (FIRI)	HbA1c < 6.50%	178	109.29	19,453.50	0.145
	HbA1c > 6.50%	46	124.92	5746.50	
	Total	224			
Glucose insulin ratio (G/I ratio)	HbA1c < 6.50%	178	106.99	19,044.00	*0.012*
	HbA1c > 6.50%	46	133.83	6156.00	
	Total	224			

The values in italic shows significant differences

## Discussion

Our study probably is the pioneer regional study in terms of introducing HOMA-TG index to include the effects of insulin related effects on glucose and triglycerides and thus went beyond the traditional insulin-glucose association concept. There is no denial or contrast in terms of understanding the association of lipid metabolism and insulin resistance as highlighted well in introduction and various other studies [11, 19, 20], but not much effort has been in place to associate this link as a mathematical model at least in our set up. The HOMA-TG index has shown the highest AUC for diagnosing metabolic syndrome, highest correlation with lipid and anthropometric indices. We also establish that HOMA-TG index results were significantly between subjects with or without diabetes. Literature presenting similar models or equations incorporating insulin, glucose and lipids are limited. However fat indices have been advocated as surrogate markers for insulin resistance. Liu et al. [21] in rural Chinese study has advocated % body fat to be useful marker for metabolic syndrome. Similarly, certain mathematical models have also been developed which have been considered as a bridge between lipid metabolism and insulin resistance using adiponectin like ADMET [22, 23]. However, we could not establish from literature search about any study suggesting an equation with insulin, glucose and lipid to assess insulin resistance; however, Pearson et al. and Pratt et al. did include some work pertaining to lipid-glucose and insulin work up and one mathematical modelling study relevant to current debate which could be relevant in some way to our established data [12, 13].

Next to establishing utility of HOMA-TG index, we observed QUICKI and G/I ratio to be least effective with lowest AUC in ROC curve analysis. This data contrasts to some of the previous finding as demonstrated by Pastucha et al. [24] where the researchers identified insulin resistance in 86% of children in comparison to 56% by HOMAIR. Similarly, Bahijri et al. [25] who found over estimation of insulin resistance by using QUICKI equation than HOMAIR. However, using free fatty acids with QUICKI as M-QUCIKI resulted in better results in this study and also by the study by Perseghin et al. which further highlights the utility of adding a lipid parameter, especially triglyceride or free fatty acids in the equation [23, 24]. The fact from the above studies highlights one aspect that QUICKI overestimates subjects with high insulin resistance and vice versa. Our study did not have many subjects known to have high grade insulin resistance, so one possibility of QUICKI not performing better at low resistance can be considered as one reason as also highlighted above by Bahijri et al. However, further studies are warranted to confirm this aspect. Finally, Keskin et al. [25] have demonstrated that HOMAIR index is better than both QUICKI and G/I ratio. Similarly, Vaccaro et al. in a large sample size study (n = 2731) have not found QUICKI or revised QUICKI methods to be better than HOMA indices in diagnosis of insulin resistance [26]. While time will confirm the reason behind data variability, but it seems that both studies by Keskin et al. and Vaccaro et al. had either pediatric population with yet to develop high insulin resistance or non-diabetic subjects where chances of underlying raised insulin resistance was minimal like our study thus demonstrated poor performance of QUICKI in comparison to HOMAIR index. However, the authors could probably establish that QUICKI may be more specific to perform better with high-grade insulin resistance but at the cost of compromised sensitivity and late detection.

Another dogma which has prevailed is the newer HOMAIR version i.e., HOMA2 index but we could not establish this index as more efficient in diagnosing metabolic syndrome. More so the correlation of HOMA2 index has shown no superiority in terms of correlation HOMAIR index with lipid indices or anthropometric parameters. In this regard the Brazilian Metabolic Syndrome Study (BRAMS) has demonstrated slightly higher AUC in ROC analysis for traditional HOMAIR than for the newly developed HOMA2 index [27]. The reasons may be related to imprecision but the large sample size reduce the likeliness of this possibility. Secondly, we as author possibly establish that HOMAIR simply includes the variability which may have resulted from pathology specific to patients and thus were demonstrating higher AUC values than HOMA2 which fixes out the imprecision resulting from biological variation. We also interpret that inter-individual variation due to biological variability is important and is better covered by simpler HOMAIR index. Similar ROC curve data supporting our finding i.e., higher AUC for original HOMAIR method in comparison to HOMA2 has been demonstrated by Mojiminiyi et al. [28–30].

It is pertinent to understand the limitations of this study: Firstly, our study was cross-sectional study carried out in a hospital setting based non-probability sampling and not a validation study, therefore the research work was meant to raise more question and highlighted a follow up “validation study”. The findings must therefore be interpreted in the same perspective. Secondly, there is a definitive possibility of type-II statistical error due to small sample size with underlying reasons linked to resource limitations. Thirdly, some part of the data was non-parametric as can be seen by higher standard deviation therefore we had to apply non-parametric tests. Lastly, based upon cross-sectional design and hospital setting the study needs to replicated in multi-central trial in randomized controlled format possibly as an epidemiological set up to confirm or refute our findings.

Our study has important clinical implication as very few studies have been able to incorporate the combined effect of insulin on both carbohydrate and fat metabolism in a mathematical model at least not in this part of the world. Addition of triglyceride to the HOMA equation improves the efficiency of the equation not only as a diagnostic test but also in the prediction of diabetes. We were also able to establish higher correlation of our suggested HOMA-TG index signifying that it can improve the yield beyond just insulin-glucose axis. Furthermore, the study has provided a mathematical solution to enhance the beta cell functional evaluation in terms of including the very significant role of insulin fat metabolism. However, we also recommend that the equation may be further explored by carrying out a large-scale study.

## Conclusion

“HOMA-TG index” has shown the highest AUC for diagnosing metabolic syndrome along with higher correlation with lipid markers and anthropometric indices in comparison to other steady-state insulin resistance markers. Furthermore, QUICKI and G/I ratio showed the lowest AUC for detection of metabolic syndrome.

## Data Availability

SPSS data outputs can be made available if requested.

## Abbreviations

OGTT: oral glucose tolerance test
MINMOD: minimally model analysis of frequently sampled intravenous glucose tolerance test
IGI: Insulinogenic index
FSIVGTT: frequently sampled intravenous glucose tolerance test
HOMAIR: Homeostasis Model Assessment for Insulin Resistance
GI ratio: glucose insulin ratio
QUICKI: Quantitative Insulin Sensitivity Check Index
FIRI: Fasting Insulin Resistance Index
